# Among the "Light" Patients at the London

**Published:** 1903-06-27

**Authors:** 


### HOSPITAL ADMINISTRATION.

### CONSTRUCTION AND ECONOMICS.

### AMONG THE "LIGHT" PATIENTS AT THE LONDON.

THROUGH the main building, past some temporary wards, across a corner of the garden, through a corridor, along a gallery which overlooks an out-patient department, and at last one reaches the door above which is incribed, "Queen Alexandra's Light Department." Inside there is light in abundance, but filtered through ground glass windows so that there is no glare. On one side of the entrance is a dressing-room for the nurses, where they don their big linen overalls before beginning to work. At the further end of the great room opens the apartment where patients await their turn and where the sister in charge marks with a soft blue pencil the part to be treated by the Finsen light at the immediate séance. In the light-room the thing which at once catches the eye is the Finsen apparatus itself—the lamp presented by Queen Alexandra, and a duplicate given by Mr. Alfred Harmsworth, with their exaggerated scarlet lamp-shades, from which the polished lamps project like some mysterious arrangement of telescopes, and beneath these the couches on which the patients lie while the rays are being turned on to their sores. At intervals round the walls are some of the "French" lamps, with the chairs for the patients to sit in when treated by that method. At either end of the room is a row of basins and taps for the nurses to cleanse their hands before and after applying the treatment.

It is an interesting sight to watch the patients gathering between séances to be ready for their turn. They form a coterie by themselves, these patients. Gathered from far and near, and living in any quarter of London for 23 hours of the day, they invariably pass the remaining hour together, and starting from the common ground of the disease from which all suffer, they develop a peculiar intimacy, which goes on for months. For the treatment is a long process. "How long have you been under treatment?" a visitor asks one and another. "Six months," "a year," "a year and seven months," as the case may be, is the answer. Those who have been longest under treatment encourage the new-comers, showing what the light has done for them. For though to an outsider the faces of almost all seem horribly scarred, the sufferers themselves know how much worse their condition was before they came to the hospital. Therefore they are, for the most part, a cheerful folk. They have, for one thing, companionship in misfortune. In their own homes they were isolated through their disfigurement, and their often repulsive appearance made strangers avoid them. But here they find others like themselves: some better, some worse, but all able to understand and sympathise with their feelings. But if this were all, community in suffering would only deepen the despair of these patients. But they have hope for themselves and for their fellows, and therefore they are cheerful.

The idiosyncrasies of the various patients come out as one watches them. A pretty girl, whose case is being taken in time, for there is only one great crimson spot on her cheek, comes in, removes her hat, arranges her hair before a mirror hung conveniently on the wall, and passing to a chair with a smile and a nod to some who are already there, buries herself in a magazine which she has brought with her. A young man, faultlessly attired, enters a little later. With business-like accuracy he goes to the couch where he is to lie during his séance, and spreads over the pillow the clean towel with which each patient is provided, so that one may not pass on contagion to the other. Then he arranges the stool on which his nurse is to sit while she holds the glass disc upon his face and the smaller one on which her feet will rest. The older women have brought their needlework with them. One has a basket by her filled with calico cut out for various garments—a busy woman, who threads her needle even while she talks to you. Another has laid down her crochet-needle for the moment, and immediately a girl who is nearly well dances away with it, pretending to steal the work, and then measuring how many yards her companion has done since she began to come to the hospital. She is very bright, this girl, overflowing with high spirits and harmless mischief. Outsiders might pity her still, with great part of her face scarred as if by a severe burn, and only the upper part of her nose intact. But she knows how much worse she has been. The nose had been eaten away by the disease before she came to the hospital, and where the seared but sound skin now is, was a mass of purulent crusts. Soon she will go out with the disease eradicated, and as for the bit of the nose that is missing, is there not in sister's drawer a photograph of one of her predecessors, who by means of a celluloid nose has achieved a comparatively attractive appearance! There is a more remarkable case still of a gentleman who came all the way from New Zealand to undergo the treatment, and with whom the disease was so far advanced that the greater part of the nose and all the upper lip were gone, but who now presents, in his photograph, a positively handsome appearance with an artificial nose and moustache. Looking at the picture of this man as he was when he came and as he looked when he went away, one can well believe that he has never ceased to sing at the Antipodes the praises of the Finsen light.

Twelve patients can be treated at a time. Eleven have come, had the spots to be treated marked, and gone to take their places. Twelve nurses have entered, donned their linen overalls, and the dark glasses which preserve their eyes from the intense light necessary for the cure of lupus. One nurse is still free; one middle-aged woman is still sitting in the preparation-room, but she has let her sewing drop, and is looking eagerly at the sister. "Yes," says the latter, after waiting for a little, "we can take you." Then she explains to the visitor that this poor soul has already had her séance for the day, but hers is a very bad case, and so she waits in the hope that, as sometimes happens, a patient may fail to come, and she may have another. This has happened to-day, and she goes eagerly to take the vacant place. She has cause to be anxious for extra treatment, for, as the sister explains with regret, while one patch is being cured the disease is spreading in another. No wonder that she is grateful for another hour of the cleansing rays. This is a case in which the Queen is greatly interested. "You remember when the Queen came and spoke to you?" asks the sister.

"Yes, sister, but I wasn't able to answer her," says the patient.

"No, it was when your lip was so bad that you could not speak," the sister explains; but perhaps there was shyness as well as disease to account for the silence with which the Royal lady's inquiries were received. But behind the silence there was abundant gratitude.

So all the chairs and couches are filled, and the period of treatment has begun. As the patients lie they see, painted in bold characters on the sides of the beams that support the ceiling, the words, "Nothing like Perseverance." It is a quotation from a letter of the Royal Foundress. The treatment is long and tiresome, and the disease does its best to keep pace with the cure. But "there's nothing like perseverance." In this as in all things, the light will in the end prevail. There are 112 patients being treated at the London Hospital alone, and 200 waiting to gain admittance. Let them not go away in despair. "Nothing like perseverance." Installations of the Finsen light have already been placed in several provincial, Scotch, and Irish hospitals, and everywhere there are more patients than can be received. More lights are wanted. But every cure is one of the best advertisements the treatment can have. As the work is done, the means for more work will be forthcoming. Growth is the law of life. The hospital officials and the hospital supporters mnst go on with their work. "Nothing like perseverance."

